# Poly[μ_2_-benzene-1,3-dicarboxyl­ato-κ^2^ *O*:*O*′-μ_2_-1,3-di-4-pyridylpropane-κ^2^ *N*:*N*′-zinc(II)]

**DOI:** 10.1107/S1600536808001621

**Published:** 2008-01-23

**Authors:** Jin-Feng Huang, Yu-Mei Dai, Jian-Rong Lin, Hui Lin, En Tang

**Affiliations:** a College of Chemistry and Materials, Fujian Normal University, Fuzhou 350007, People’s Republic of China; b State Key Laboratory of Structural Chemistry, Fujian Institute of Research on the Structure of Matter, Chinese Academy of Sciences, Fuzhou, Fujian 350002, People’s Republic of China; c Conjugate and Medicinal Chemistry Laboratory, Department of Radiology, Brigham and Women’s Hospital and Harvard Medical School, Boston, MA 02115, USA

## Abstract

The title compound, [Zn(C_8_H_4_O_4_)(C_13_H_14_N_2_)]_*n*_, was obtained by the hydro­thermal reaction of Zn(OAc)_2_·H_2_O with 1,3-di-4-pyridylpropane (bpp) and isophthalic acid (H_2_ip). The Zn^II^ ion is coordinated by two bpp and two ip ligands in a distorted tetra­hedral environment. Each ligand coordinates in a bridging mode to connect Zn^II^ ions into a three-dimensional diamondoid-type structure.

## Related literature

For related literature, see: Dai *et al.* (2005 ▶); Evans *et al.* (1999 ▶); Tang *et al.* (2004 ▶); Fujita *et al.* (1994 ▶).
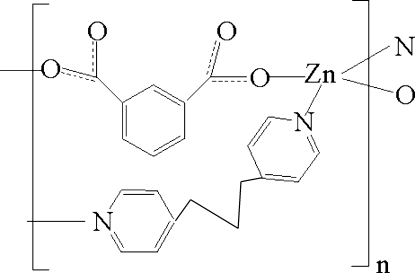


## Experimental

### 

#### Crystal data

[Zn(C_8_H_4_O_4_)(C_13_H_14_N_2_)]
*M* *_r_* = 427.76
Monoclinic, 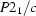

*a* = 11.0418 (13) Å
*b* = 11.1924 (14) Å
*c* = 16.8687 (17) Å
β = 115.249 (7)°
*V* = 1885.5 (4) Å^3^
*Z* = 4
Mo *K*α radiation
μ = 1.33 mm^−1^
*T* = 293 (2) K
0.30 × 0.20 × 0.10 mm

#### Data collection

Bruker SMART CCD diffractometer
Absorption correction: multi-scan (*SADABS*; Sheldrick, 1996 ▶) *T*_min_ = 0.733, *T*_max_ = 0.875
14111 measured reflections
4328 independent reflections
3887 reflections with *I* > 2σ(*I*)
*R*_int_ = 0.020

#### Refinement

*R*[*F* ^2^ > 2σ(*F* ^2^)] = 0.040
*wR*(*F* ^2^) = 0.115
*S* = 1.04
4328 reflections
254 parameters
H-atom parameters constrained
Δρ_max_ = 0.73 e Å^−3^
Δρ_min_ = −0.76 e Å^−3^

### 

Data collection: *SMART* (Siemens, 1996 ▶); cell refinement: *SAINT* (Siemens, 1997 ▶); data reduction: *SAINT*; program(s) used to solve structure: *SHELXTL* (Sheldrick, 2008 ▶); program(s) used to refine structure: *SHELXTL*; molecular graphics: *SHELXTL*; software used to prepare material for publication: *SHELXTL*.

## Supplementary Material

Crystal structure: contains datablocks I, global. DOI: 10.1107/S1600536808001621/lh2583sup1.cif

Structure factors: contains datablocks I. DOI: 10.1107/S1600536808001621/lh2583Isup2.hkl

Additional supplementary materials:  crystallographic information; 3D view; checkCIF report

## Figures and Tables

**Table:** 

Zn1—O2	1.9511 (17)
Zn1—O4^i^	1.9621 (17)
Zn1—N2	2.041 (2)
Zn1—N1	2.051 (2)

**Table:** 

O2—Zn1—O4^i^	101.92 (7)
O2—Zn1—N2	114.19 (8)
O4^i^—Zn1—N2	122.01 (8)
O2—Zn1—N1	109.01 (8)
O4^i^—Zn1—N1	100.98 (8)
N2—Zn1—N1	107.55 (8)

Symmetry code: (i) 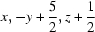.

**Fig. 1.**
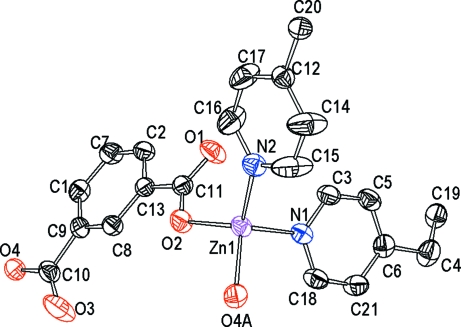
The asymmetric unit showing 30% probability displacement ellipsoids. A symmetry related O atom is shown to complete the tetrahedral coordination [symmetry code: (A) x, 5/2 - y, 1/2 + z]. H atoms are not shown.

**Fig. 2.**
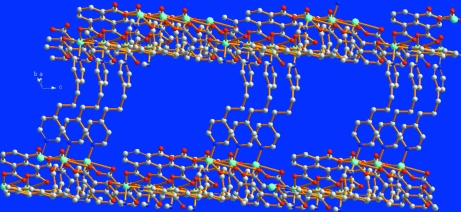
Part of the crystal structure of the title compound.

## supplementary crystallographic information

### Comment

A large family of coordination polymers has been developed recently owing to their potential applications as functional solid materials and their intriguing architectures or topologies (Evans *et al.*, 1999; Fujita *et al.*, 1994). It is now well understood that the hydrothermal crystallization of metal centers with multidentate N– or O-donor ligands, which possess more rich coordination sites and a wide variety of shapes to facilitate the formation of various networks, is one of the useful approaches to assembly desired new materials. An impressive literature of one-, two- and three-dimensional frameworks based on these ligands (Dai *et al.*, 2005,Tang *et al.*, 2004) with various structural motifs, such as helical, brick wall, ladder, honeycomb, square grid, parquet, and diamondoid, have been reported to date. Here we report the synthesis and crystal structure of the title compound (I).

In (I) [Fig. 1] each Zn^II^ ion coordinates to two pyridine N atoms of two bpp ligands and two carboxylate groups of two ip ligands, in monodentate modes, giving a distorted tetrahedral coordination environment. Both bpp and ip ligands coordinate in bridging modes to for a three-dimensional diamondoid structure with Zn···Zn separations of 9.425 and 12.745Å and formimg cavities within the structure (Fig. 2).

### Experimental

A mixture of Zn(Ac)_2_ H_2_O (1.00 mmol, 0.22 g), bpp (1.00 mmol, 0.19 g), H_2_ip (1.00 mmol, 0.16 g) and H_2_O (15 ml) was vigorous stirring until the pH was adjusted to 6 by adding 10% NaOH. This mixture was heated at 433 K for 3 days in a sealed 25 ml Teflon-lined stainless steel vessel under autogenous pressure. After cooling to room temperature at 50 K h^-1^, orange prism-shaped crystals were isolated, which were washed with ethanol and dried in air.

### Refinement

H atoms were positioned geometrically and refined using a riding model [C—H 0.93–0.97Å and *U*_iso_(H) = 1.2*U*_eq_(C)].

### Crystal data

**Table:**

[Zn(C_8_H_4_O_4_)(C_13_H_14_N_2_)]	*F*_000_ = 880
*M**_r_* = 427.76	*D*_x_ = 1.507 Mg m^−^^3^
Monoclinic, *P*2_1_/*c*	Mo *K*α radiation λ = 0.71073 Å
Hall symbol: -P 2ybc	Cell parameters from 4994 reflections
*a* = 11.0418 (13) Å	θ = 3.2–27.5º
*b* = 11.1924 (14) Å	µ = 1.33 mm^−^^1^
*c* = 16.8687 (17) Å	*T* = 293 (2) K
β = 115.249 (7)º	Prism, orange
*V* = 1885.5 (4) Å^3^	0.30 × 0.20 × 0.10 mm
*Z* = 4	

### Data collection

**Table:**

Bruker SMART CCD diffractometer	4328 independent reflections
Radiation source: fine-focus sealed tube	3887 reflections with *I* > 2σ(*I*)
Monochromator: graphite	*R*_int_ = 0.020
*T* = 293(2) K	θ_max_ = 27.5º
ω scans	θ_min_ = 3.2º
Absorption correction: multi-scan(SADABS; Sheldrick, 1996)	*h* = −14→14
*T*_min_ = 0.733, *T*_max_ = 0.875	*k* = −11→14
14111 measured reflections	*l* = −21→21

### Refinement

**Table:**

Refinement on *F*^2^	Secondary atom site location: difference Fourier map
Least-squares matrix: full	Hydrogen site location: inferred from neighbouring sites
*R*[*F*^2^ > 2σ(*F*^2^)] = 0.040	H-atom parameters constrained
*wR*(*F*^2^) = 0.115	*w* = 1/[σ^2^(*F*_o_^2^) + (0.0695*P*)^2^ + 1.1112*P*] where *P* = (*F*_o_^2^ + 2*F*_c_^2^)/3
*S* = 1.04	(Δ/σ)_max_ = 0.002
4328 reflections	Δρ_max_ = 0.73 e Å^−^^3^
254 parameters	Δρ_min_ = −0.76 e Å^−^^3^
Primary atom site location: structure-invariant direct methods	Extinction correction: none

### Special details

**Table:**

Geometry. All e.s.d.'s (except the e.s.d. in the dihedral angle between two l.s. planes) are estimated using the full covariance matrix. The cell e.s.d.'s are taken into account individually in the estimation of e.s.d.'s in distances, angles and torsion angles; correlations between e.s.d.'s in cell parameters are only used when they are defined by crystal symmetry. An approximate (isotropic) treatment of cell e.s.d.'s is used for estimating e.s.d.'s involving l.s. planes.
Refinement. Refinement of *F*^2^ against ALL reflections. The weighted *R*-factor *wR* and goodness of fit *S* are based on *F*^2^, conventional *R*-factors *R* are based on *F*, with *F* set to zero for negative *F*^2^. The threshold expression of *F*^2^ > σ(*F*^2^) is used only for calculating *R*-factors(gt) *etc*. and is not relevant to the choice of reflections for refinement. *R*-factors based on *F*^2^ are statistically about twice as large as those based on *F*, and *R*- factors based on ALL data will be even larger.

### Fractional atomic coordinates and isotropic or equivalent isotropic displacement parameters (Å^2^)

**Table:**

	*x*	*y*	*z*	*U*_iso_*/*U*_eq_	
Zn1	0.21026 (3)	1.06213 (2)	0.850451 (16)	0.03187 (11)	
O1	0.1788 (2)	0.88899 (17)	0.71665 (12)	0.0485 (5)	
O2	0.2385 (2)	1.07900 (15)	0.74443 (11)	0.0405 (4)	
O3	0.2086 (3)	1.3428 (2)	0.50730 (17)	0.0828 (9)	
O4	0.22045 (18)	1.27148 (16)	0.38941 (11)	0.0412 (4)	
N1	0.0122 (2)	1.02485 (18)	0.81573 (13)	0.0346 (4)	
N2	0.3207 (2)	0.92958 (18)	0.93261 (13)	0.0379 (5)	
C1	0.1641 (2)	1.0385 (2)	0.43263 (15)	0.0375 (5)	
H1A	0.1559	1.0490	0.3759	0.045*	
C2	0.1551 (2)	0.9102 (2)	0.54416 (16)	0.0354 (5)	
H2A	0.1387	0.8354	0.5616	0.042*	
C3	−0.0341 (3)	0.9129 (2)	0.81017 (17)	0.0388 (5)	
H3A	0.0255	0.8498	0.8201	0.047*	
C4	−0.4016 (3)	0.9562 (3)	0.7578 (2)	0.0491 (7)	
H4A	−0.4054	0.9442	0.8137	0.059*	
H4B	−0.4533	1.0272	0.7313	0.059*	
C5	−0.1657 (3)	0.8869 (2)	0.79050 (16)	0.0410 (5)	
H5A	−0.1931	0.8078	0.7875	0.049*	
C6	−0.2578 (2)	0.9791 (2)	0.77504 (15)	0.0370 (5)	
C7	0.1420 (3)	0.9271 (2)	0.45968 (17)	0.0407 (6)	
H7A	0.1182	0.8632	0.4207	0.049*	
C8	0.2136 (2)	1.1160 (2)	0.57552 (14)	0.0333 (5)	
H8A	0.2380	1.1798	0.6146	0.040*	
C9	0.1985 (2)	1.1341 (2)	0.49008 (14)	0.0332 (5)	
C10	0.2109 (3)	1.2590 (2)	0.46194 (16)	0.0403 (5)	
C11	0.2035 (2)	0.9872 (2)	0.69431 (14)	0.0322 (5)	
C12	0.4479 (2)	0.7383 (2)	1.04535 (15)	0.0353 (5)	
C13	0.1929 (2)	1.0050 (2)	0.60318 (14)	0.0302 (4)	
C14	0.3843 (5)	0.8270 (3)	1.0680 (2)	0.0765 (12)	
H14A	0.3829	0.8252	1.1227	0.092*	
C15	0.3220 (5)	0.9198 (3)	1.0112 (2)	0.0780 (13)	
H15A	0.2790	0.9782	1.0290	0.094*	
C16	0.3822 (3)	0.8432 (3)	0.91049 (19)	0.0596 (9)	
H16A	0.3829	0.8469	0.8556	0.072*	
C17	0.4451 (3)	0.7481 (3)	0.96411 (18)	0.0575 (8)	
H17A	0.4862	0.6900	0.9446	0.069*	
C18	−0.0769 (3)	1.1138 (2)	0.79964 (17)	0.0413 (5)	
H18A	−0.0472	1.1921	0.8027	0.050*	
C19	−0.4686 (3)	0.8497 (2)	0.69901 (16)	0.0424 (6)	
H19A	−0.4147	0.7790	0.7231	0.051*	
H19B	−0.5554	0.8365	0.6990	0.051*	
C20	0.5127 (2)	0.6335 (2)	1.10512 (16)	0.0393 (5)	
H20A	0.5996	0.6190	1.1055	0.047*	
H20B	0.4582	0.5630	1.0811	0.047*	
C21	−0.2100 (3)	1.0941 (2)	0.77886 (18)	0.0429 (6)	
H21A	−0.2682	1.1586	0.7673	0.051*	

### Atomic displacement parameters (Å^2^)

**Table:**

	*U*^11^	*U*^22^	*U*^33^	*U*^12^	*U*^13^	*U*^23^
Zn1	0.03897 (18)	0.03055 (17)	0.02643 (16)	−0.00058 (10)	0.01427 (12)	0.00286 (9)
O1	0.0735 (13)	0.0335 (9)	0.0400 (9)	−0.0039 (9)	0.0257 (9)	0.0072 (8)
O2	0.0602 (11)	0.0360 (9)	0.0325 (8)	−0.0046 (8)	0.0266 (8)	−0.0023 (7)
O3	0.166 (3)	0.0358 (11)	0.0722 (15)	−0.0180 (14)	0.0749 (18)	−0.0039 (11)
O4	0.0501 (10)	0.0401 (9)	0.0381 (9)	0.0086 (8)	0.0233 (8)	0.0138 (7)
N1	0.0386 (10)	0.0289 (9)	0.0346 (9)	−0.0028 (8)	0.0139 (8)	−0.0002 (8)
N2	0.0436 (11)	0.0367 (11)	0.0306 (10)	0.0021 (8)	0.0131 (9)	0.0055 (8)
C1	0.0402 (12)	0.0450 (13)	0.0271 (10)	0.0023 (10)	0.0143 (9)	0.0005 (10)
C2	0.0417 (12)	0.0290 (11)	0.0362 (11)	−0.0001 (9)	0.0173 (10)	0.0000 (9)
C3	0.0459 (13)	0.0282 (11)	0.0433 (13)	0.0008 (10)	0.0198 (11)	0.0017 (10)
C4	0.0458 (14)	0.0557 (17)	0.0506 (15)	−0.0129 (12)	0.0250 (13)	−0.0232 (13)
C5	0.0525 (14)	0.0300 (12)	0.0417 (13)	−0.0090 (10)	0.0213 (11)	−0.0047 (10)
C6	0.0410 (12)	0.0406 (13)	0.0301 (10)	−0.0072 (10)	0.0160 (9)	−0.0095 (10)
C7	0.0514 (15)	0.0356 (13)	0.0346 (12)	−0.0032 (10)	0.0180 (11)	−0.0090 (10)
C8	0.0411 (12)	0.0304 (11)	0.0301 (10)	−0.0019 (9)	0.0167 (9)	−0.0020 (9)
C9	0.0369 (11)	0.0338 (12)	0.0306 (10)	0.0009 (9)	0.0160 (9)	0.0043 (9)
C10	0.0510 (14)	0.0363 (13)	0.0370 (12)	−0.0009 (10)	0.0221 (11)	0.0055 (10)
C11	0.0365 (11)	0.0316 (11)	0.0301 (10)	0.0039 (9)	0.0157 (9)	0.0031 (9)
C12	0.0342 (11)	0.0348 (12)	0.0343 (11)	0.0005 (9)	0.0121 (9)	0.0042 (9)
C13	0.0324 (10)	0.0300 (11)	0.0296 (10)	0.0029 (8)	0.0146 (8)	0.0019 (8)
C14	0.145 (4)	0.0503 (18)	0.0402 (15)	0.042 (2)	0.046 (2)	0.0145 (13)
C15	0.148 (4)	0.0481 (17)	0.0414 (16)	0.046 (2)	0.044 (2)	0.0115 (13)
C16	0.0671 (19)	0.078 (2)	0.0407 (14)	0.0318 (17)	0.0300 (14)	0.0196 (14)
C17	0.0651 (18)	0.069 (2)	0.0436 (14)	0.0355 (16)	0.0278 (14)	0.0143 (14)
C18	0.0454 (13)	0.0274 (12)	0.0484 (14)	−0.0035 (10)	0.0175 (11)	−0.0010 (10)
C19	0.0398 (13)	0.0458 (14)	0.0429 (13)	−0.0119 (11)	0.0190 (11)	−0.0146 (11)
C20	0.0399 (13)	0.0367 (13)	0.0417 (12)	0.0067 (10)	0.0179 (10)	0.0076 (10)
C21	0.0429 (14)	0.0339 (12)	0.0493 (14)	0.0026 (11)	0.0172 (12)	−0.0044 (11)

### Geometric parameters (Å, °)

**Table:**

Zn1—O2	1.9511 (17)	C5—H5A	0.9300
Zn1—O4^i^	1.9621 (17)	C6—C21	1.383 (4)
Zn1—N2	2.041 (2)	C7—H7A	0.9300
Zn1—N1	2.051 (2)	C8—C9	1.393 (3)
O1—C11	1.230 (3)	C8—C13	1.381 (3)
O2—C11	1.281 (3)	C8—H8A	0.9300
O3—C10	1.218 (3)	C9—C10	1.501 (3)
O4—C10	1.279 (3)	C11—C13	1.504 (3)
O4—Zn1^ii^	1.9621 (17)	C12—C14	1.361 (4)
N1—C18	1.343 (3)	C12—C17	1.362 (3)
N1—C3	1.342 (3)	C12—C20	1.513 (3)
N2—C16	1.323 (4)	C14—C15	1.381 (4)
N2—C15	1.324 (4)	C14—H14A	0.9300
C1—C9	1.384 (3)	C15—H15A	0.9300
C1—C7	1.384 (4)	C16—C17	1.377 (4)
C1—H1A	0.9300	C16—H16A	0.9300
C2—C7	1.383 (3)	C17—H17A	0.9300
C2—C13	1.392 (3)	C18—C21	1.376 (4)
C2—H2A	0.9300	C18—H18A	0.9300
C3—C5	1.376 (4)	C19—C20^iii^	1.519 (3)
C3—H3A	0.9300	C19—H19A	0.9700
C4—C19	1.524 (3)	C19—H19B	0.9700
C4—C6	1.509 (4)	C20—C19^iv^	1.519 (3)
C4—H4A	0.9700	C20—H20A	0.9700
C4—H4B	0.9700	C20—H20B	0.9700
C5—C6	1.393 (4)	C21—H21A	0.9300
			
O2—Zn1—O4^i^	101.92 (7)	C1—C9—C10	122.2 (2)
O2—Zn1—N2	114.19 (8)	O3—C10—O4	123.3 (2)
O4^i^—Zn1—N2	122.01 (8)	O3—C10—C9	119.3 (2)
O2—Zn1—N1	109.01 (8)	O4—C10—C9	117.4 (2)
O4^i^—Zn1—N1	100.98 (8)	O1—C11—O2	123.9 (2)
N2—Zn1—N1	107.55 (8)	O1—C11—C13	120.0 (2)
C11—O2—Zn1	113.99 (14)	O2—C11—C13	116.04 (19)
C10—O4—Zn1^ii^	114.07 (17)	C14—C12—C17	115.5 (2)
C18—N1—C3	117.0 (2)	C14—C12—C20	122.2 (2)
C18—N1—Zn1	120.43 (17)	C17—C12—C20	122.3 (2)
C3—N1—Zn1	122.55 (17)	C8—C13—C2	119.1 (2)
C16—N2—C15	115.7 (2)	C8—C13—C11	120.8 (2)
C16—N2—Zn1	124.72 (18)	C2—C13—C11	120.0 (2)
C15—N2—Zn1	119.2 (2)	C12—C14—C15	121.2 (3)
C9—C1—C7	120.0 (2)	C12—C14—H14A	119.4
C9—C1—H1A	120.0	C15—C14—H14A	119.4
C7—C1—H1A	120.0	N2—C15—C14	123.1 (3)
C7—C2—C13	120.1 (2)	N2—C15—H15A	118.4
C7—C2—H2A	120.0	C14—C15—H15A	118.4
C13—C2—H2A	120.0	N2—C16—C17	123.7 (2)
N1—C3—C5	123.1 (2)	N2—C16—H16A	118.1
N1—C3—H3A	118.5	C17—C16—H16A	118.1
C5—C3—H3A	118.5	C16—C17—C12	120.8 (3)
C19—C4—C6	115.9 (2)	C16—C17—H17A	119.6
C19—C4—H4A	108.3	C12—C17—H17A	119.6
C6—C4—H4A	108.3	N1—C18—C21	122.9 (2)
C19—C4—H4B	108.3	N1—C18—H18A	118.5
C6—C4—H4B	108.3	C21—C18—H18A	118.5
H4A—C4—H4B	107.4	C20^iii^—C19—C4	113.2 (2)
C3—C5—C6	120.0 (2)	C20^iii^—C19—H19A	108.9
C3—C5—H5A	120.0	C4—C19—H19A	108.9
C6—C5—H5A	120.0	C20^iii^—C19—H19B	108.9
C5—C6—C21	116.6 (2)	C4—C19—H19B	108.9
C5—C6—C4	122.3 (2)	H19A—C19—H19B	107.7
C21—C6—C4	121.1 (2)	C12—C20—C19^iv^	114.5 (2)
C2—C7—C1	120.5 (2)	C12—C20—H20A	108.6
C2—C7—H7A	119.8	C19^iv^—C20—H20A	108.6
C1—C7—H7A	119.8	C12—C20—H20B	108.6
C9—C8—C13	121.1 (2)	C19^iv^—C20—H20B	108.6
C9—C8—H8A	119.5	H20A—C20—H20B	107.6
C13—C8—H8A	119.5	C18—C21—C6	120.4 (2)
C8—C9—C1	119.2 (2)	C18—C21—H21A	119.8
C8—C9—C10	118.4 (2)	C6—C21—H21A	119.8

Symmetry codes: (i) *x*, −*y*+5/2, *z*+1/2; (ii) *x*, −*y*+5/2, *z*−1/2; (iii) *x*−1, −*y*+3/2, *z*−1/2; (iv) *x*+1, −*y*+3/2, *z*+1/2.
